# Etymologia: Creutzfeldt-Jakob Disease

**DOI:** 10.3201/eid2306.ET2306

**Published:** 2017-06

**Authors:** Ronnie Henry, Frederick A. Murphy

**Keywords:** Creutzfeldt-Jakob disease, CJD, variant Creutzfeldt-Jakob disease vCJD, Walther Spielmeyer, prions and related diseases, prion, meningitis/encephalitis, Hans Gerhard Creutzfeldt, Alfons Maria Jakob

## Creutzfeldt-Jakob [croytsʹfelt-jakʺob] Disease

In 1920, German neuropathologist Alfons Maria Jakob (Figure) described a series of 6 patients with spasticity and progressive dementia associated with neural degeneration. Shortly thereafter, in 1921, another German neuropathologist, Hans Gerhardt Creutzfeldt (Figure), independently published a similar case. Jakob gave credit to Creutzfeldt for describing the syndrome first, without realizing he had also uncovered the new syndrome. Walther Spielmeyer first used the term “Creutzfeldt-Jakob disease” (CJD) in 1922. CJD occurs worldwide as a rare, sporadic disease, with genetic and iatrogenic forms (Figure). A zoonotic form, variant CJD (vCJD), is caused by infection with a prion derived from bovines and occurs predominantly in the United Kingdom.

**Figure:**
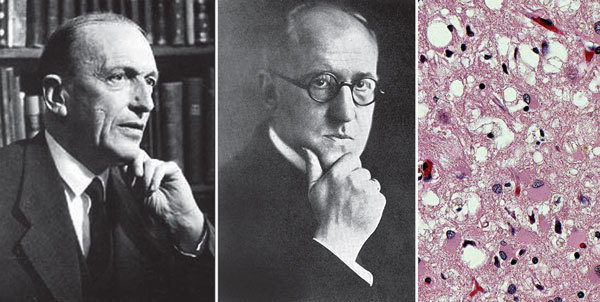
(L–R) Hans Gerhard Creutzfeldt (1885–1964; Alfons Maria Jakob (1884–1931); Creutzfeldt–Jakob disease, cerebrum hematoxylin and eosin staining showing spongiform encephalophathy. Images reproduced from Foundations of Virology, 2012, courtesy Frederick A. Murphy.

This issue of Emerging Infectious Diseases’ long-running *Etymologia* series is dedicated to the memory of Richard T. Johnson, MD (1931–2015), the leading prion disease authority in the United States for many years and great friend of CDC’s infectious disease programs, so many of which involve central nervous system disorders.
